# The Weak 3D Topological Insulator Bi_12_Rh_3_Sn_3_I_9_

**DOI:** 10.1002/chem.202001953

**Published:** 2020-10-04

**Authors:** Mai Lê Anh, Martin Kaiser, Madhav Prasad Ghimire, Manuel Richter, Klaus Koepernik, Markus Gruschwitz, Christoph Tegenkamp, Thomas Doert, Michael Ruck

**Affiliations:** ^1^ Faculty of Chemistry and Food Chemistry Technische Universität Dresden 01062 Dresden Germany; ^2^ Central Department of Physics Tribhuvan University Kirtipur, Kathmandu Nepal; ^3^ Leibniz IFW Dresden 01069 Dresden Germany; ^4^ Dresden Center for Computational Materials Science (DCMS) Technische Universität Dresden 01062 Dresden Germany; ^5^ Institute of Physics Technische Universität Chemnitz 09126 Chemnitz Germany; ^6^ Max Planck Institute for Chemical Physics of Solids 01187 Dresden Germany

**Keywords:** crystal growth, crystal structure, topological band gap, topological insulators, weak topological insulators

## Abstract

Topological insulators (TIs) gained high interest due to their protected electronic surface states that allow dissipation‐free electron and information transport. In consequence, TIs are recommended as materials for spintronics and quantum computing. Yet, the number of well‐characterized TIs is rather limited. To contribute to this field of research, we focused on new bismuth‐based subiodides and recently succeeded in synthesizing a new compound Bi_12_Rh_3_Sn_3_I_9_, which is structurally closely related to Bi_14_Rh_3_I_9_ – a stable, layered material. In fact, Bi_14_Rh_3_I_9_ is the first experimentally supported weak 3D TI. Both structures are composed of well‐defined intermetallic layers of _∞_^2^[(Bi_4_Rh)_3_I]^2+^ with topologically protected electronic edge‐states. The fundamental difference between Bi_14_Rh_3_I_9_ and Bi_12_Rh_3_Sn_3_I_9_ lies in the composition and the arrangement of the anionic spacer. While the intermetallic 2D TI layers in Bi_14_Rh_3_I_9_ are isolated by _∞_^1^[Bi_2_I_8_]^2−^ chains, the isoelectronic substitution of bismuth(III) with tin(II) leads to _∞_^2^[Sn_3_I_8_]^2−^ layers as anionic spacers. First transport experiments support the 2D character of this material class and revealed metallic conductivity.

## Graphical abstract

**Insulating**: Bi_12_Rh_3_Sn_3_I_9_, a weak 3D topological insulator (TI) is derived from Bi_14_Rh_3_I_9_, which was the first weakly coupling (“weak”) 3D TI; a bulk semiconductor that hosts protected electronic surface states that allow dissipation‐free electron and information transport due to the specific symmetries of its electronic band structure. In consequence of their topological properties, TIs are envisioned as promising candidates for high‐performance spin field‐effect transistors and quantum bits.

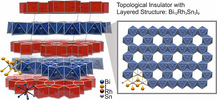

## Introduction

Large efforts have been devoted to the synthesis and to the physical fundamentals of topological insulators (TIs) to investigate their intriguing physical properties.[^1^, ^2^] While being bulk semiconductors, TIs can host protected metallic states on their surfaces. Electrons in these topologically protected surface states are sheltered against scattering. Their spin and momentum are locked orthogonally to their propagation direction.[^3^, ^4^] This effect is manifested in almost dissipation‐free electron transport. Thus, TIs are envisioned as promising candidates in the field of electronics for high‐performance spin field‐effect transistors.^[5]^ and quantum bits in quantum computing.^[6]^ The discoveries started with the investigations on the quantum Hall and the fractional quantum Hall effect, years ago. Both impressive phenomena were awarded with the Nobel Prize in Physics in 1985 and 1998, respectively, and led to comprehensive research in the following years. In 2006 and 2007, the theoretical prediction and the experimental observation of the quantum spin Hall effect in HgTe wells marked the beginning of the investigation on TIs.[^7^, ^8^] The focus was put on the electronic state, which is similar to the quantum Hall effect, but emerged due to the inherent spin‐orbit coupling instead of the external magnetic field. The existence of a quantum effect at room temperature reveals a great potential for applications and stimulated a wide search for new TIs.

**Figure 1:**
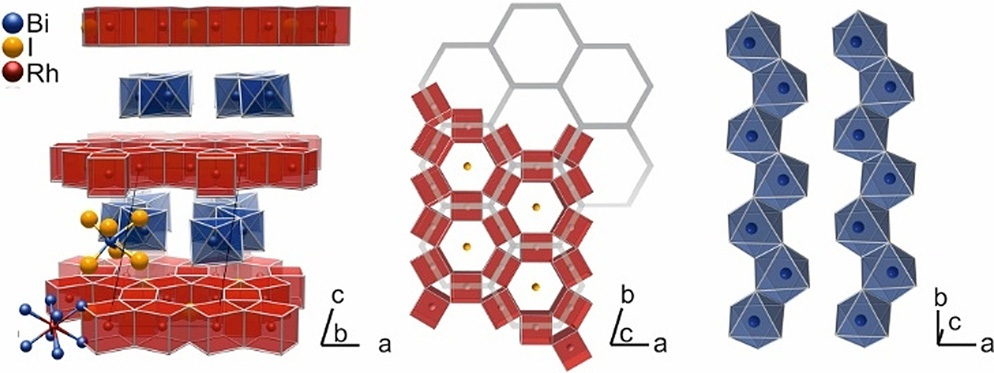
(left) Crystal structure of Bi_14_Rh_3_I_9_ (colour code: bismuth=blue, rhodium=red, iodine=orange); (middle) 2D TI layer _∞_^2^[(Bi_4_Rh)_3_I]^2+^: edge‐sharing [Bi_8/2_Rh] cubes forming a hexagonal shaped network with iodine atoms in the hexagonal prismatic voids (red‐coloured polyhedra); (right) topologically trivial spacer _∞_^1^[Bi_2_I_8_]^2−^: parallel iodido‐bismuthate chains of edge‐sharing octahedra (blue‐coloured polyhedra).

In the recent past, our group has pursued research in TIs and has contributed to the synthesis and characterization of, for example, Bi_14_Rh_3_I_9_,[^9^, ^10^, ^11^, ^12^, ^13^] Bi_*n*_TeI (*n=*1, 2),[^14^, ^15^] and MnBi_2_Te_4_.^[16]^ Among those, Bi_14_Rh_3_I_9_ is known as the first experimentally verified weak 3D TI. Its large topologically non‐trivial band gap of 210 meV is generated by strong spin‐orbit coupling (SOC) and assures that the quantum state persists at room temperature and up to the decomposition temperature of the compound. Topological edge states have been observed by scanning tunnelling microscopy on surface steps of Bi_14_Rh_3_I_9_.^[10]^ Bi_14_Rh_3_I_9_ comprises intermetallic kagome networks of rhodium‐centred bismuth cubes that share common edges forming hexagonal‐prismatic gaps, in which iodine atoms are located (Figure 1).[^9^, ^17^] As previous works have demonstrated, the main covalent bonding system is located inside the _∞_^2^[(Bi_4_Rh)_3_I]^2+^ intermetallic layer. Each of these layers was characterized as a 2D TI, if appropriate charge compensation is applied.^[12]^ These layers together with the topologically trivial anionic spacer of _∞_^1^[Bi_2_I_8_]^2−^ iodide‐bismuthate chains form an alternating stack that is weakly coupling, exhibiting a weak 3D topological character.^[9]^

Lately, a large number of subhalides based on bismuth and transition metals have been discovered.[^18^, ^19^, ^20^] Their structures typically consist of low‐dimensional intermetallic fragments with strong bonding inside and weaker, predominantly electrostatic interactions to their surroundings. Layered structures comprising the same type of intermetallic kagome nets as Bi_14_Rh_3_I_9_ are Bi_38_Pt_9_I_14_, Bi_13_Pt_3_I_7_ and Bi_12_Pt_3_I_5_.[^13^, ^21^, ^22^]

Remarkably, the intermetallic networks of several bismuth subhalides are tolerant to electronic alterations and flexible enough to provide diffusion paths for excessive mass transports. For example, single‐crystals of the metallic subiodide Bi_13_Pt_3_I_7_ were treated with *n‐*butyllithium to obtain Bi_12_Pt_3_I_5_. Interestingly, the motif of the intermetallic network still persists even after such a harsh treatment, demonstrating the stability of the net.^[23]^ Similarly, a “breathing mode” of the intermetallic framework of Bi_12_Rh_3_Cl_2_ allows a substitution of chloride ions with bismuth resulting in the transformation into the binary intermetallic compound Bi_12_Rh_3_Bi_2_=Bi_14_Rh_3_.^[19]^ The intermetallic layers of Bi_14_Rh_3_I_9_ are most likely mechanically robust and not prone to distortion, rolling‐up or furling. This opens a large field for possible structural and chemical variations, like for example, doping, de‐/intercalation reactions and changes of stacking distances. Chemical gating^[23]^ was suggested as a way to compensate the surface polarity that shifts the topological edge states away from the Fermi level in pristine samples. According to theoretical simulations based on Bi_14_Rh_3_I_9_, transition metal (TM) exchange in the series of platinum group elements affects the size and shape of the topological band gap, but the topological character generally remains intact.^[12]^ Only the _∞_^2^[(Bi_4_Rh)_3_I]^2+^ intermetallic layer was considered in the calculation to reduce the structural complexity. In the case of TM=Rh, two non‐trivial energy gaps can be found near the Fermi‐energy, hence supporting the 2D TI state. Furthermore, _∞_^2^[(Bi_4_ *X*)_3_I]^2+^ layers with *X*=Ru, Pd, Os, Ir and Pt instead of Rh were studied theoretically, showing topologically non‐trivial energy gaps for Ir‐, Pt‐ and Pd‐based layers, while layers substituted with Os and Ru remain trivial. This prediction has triggered the research interest in further weak 3D TIs.

Herein, we focus on compositional and structural manipulation of Bi_14_Rh_3_I_9_. In particular, the new phase Bi_12_Rh_3_Sn_3_I_9_ is experimentally obtained by formal substitution of bismuth by tin in the anionic spacer of Bi_14_Rh_3_I_9_. By doing so, central questions arise: Does a substitution influence the band structure drastically? Will the topological character persist? If so, we might be able to achieve tuneable compositions and topological band gaps. Moreover, this could open a pathway for the inclusion of magnetic ions and thereby contribute to the current quest for magnetic topological insulators. For a proper answer to those questions, the electronic properties of Bi_12_Rh_3_Sn_3_I_9_ were studied by density‐functional theory (DFT) based calculations. Moreover, we performed first temperature‐dependent transport experiments in order to underline the high conductivity within this layered material.

## Results and Discussion

### Thermal analysis of Bi_12_Rh_3_Sn_3_I_9_

**Figure 2:**
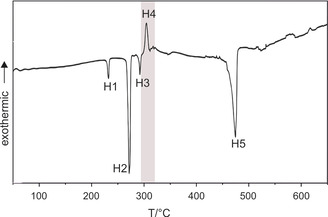
DSC data of a heated Bi, Rh, Sn, and BiI_3_ mixture in a molar ratio of 12:3:3:9 to 650 °C. The formation of the desired phase Bi_12_Rh_3_Sn_3_I_9_ is highlighted (grey area).

Insightful studies had already been devoted solely to the synthesis of Bi_14_Rh_3_I_9_ with respect to phase purity and crystal growth conditions.^[17]^ According to the previous report on the ternary Bi‐Rh‐I system, DSC studies are proven to be an effective technique to attain deeper understanding of phase formation and thermal stability. Thus, investigations in the quaternary Bi‐Rh‐Sn‐I system were performed by heating a ground mixture of Bi, Rh, Sn and BiI_3_ in a molar ratio of 12:3:3:9 in a fused silica ampule under vacuum. The DSC data shows five significant signals (Figure 2), which can be addressed to various effects (Table 1).

**Table 1:** Allocation of the DSC signals on a heated mixture of Bi, Rh, Sn and BiI_3_ (12:3:3:9) from 25 to 800 °C.

signal	*ϑ*_onset_ [°C]	effect	allocation
H1	232	endothermic	melting of Sn
H2	271	endothermic	melting of Bi
		exothermic	formation of *rt*‐Bi_2_Rh, Bi_4_I_4_
H3	292	endothermic	decomposition of Bi_4_I_4_
H4	305	exothermic	formation of Bi_12_Rh_3_Sn_3_I_9_
H5	475	endothermic	decomposition of Bi_12_Rh_3_Sn_3_I_9_

The signals were interpreted based on published data,^[17]^ and by annealing stoichiometric mixtures of the starting materials for approximately 3 h at the temperatures derived from the DSC analyses followed by an identification with X‐ray powder diffraction. The first endothermic effects, H1 at 232 °C and H2 at 271 °C, were assigned to the melting of the elements Sn^[24]^ and Bi,^[25]^ respectively. When heating the mixture iteratively to 280 °C two weakly exothermic signals appear, which can be attributed to the formation of *rt*‐Bi_2_Rh^[26]^ and Bi_4_I_4_.^[25]^ The latter decomposes at 292 °C (H3). In the next step, H4 at 305 °C, an unknown phase is formed, which was later identified as Bi_12_Rh_3_Sn_3_I_9_. Once the temperature of about 475 °C (H5) is reached, the decomposition of Bi_12_Rh_3_Sn_3_I_9_ starts gradually.

After the DSC experiments the resulting solid shows a diffraction pattern similar to Bi_14_Rh_3_I_9_. By‐products such as BiI_3_ and Bi_4_I_4_ are formed as well (Supporting Information Figure 1). Bi_12_Rh_3_Sn_3_I_9_ is not perceptively sensitive to air, but should be stored under inert gas.

Based on this study, a mixture of Bi, Rh, Sn, and BiI_3_ with the composition of the title compound was ground in a ball mill, pressed to a pill, sintered at 310 °C for several days and subsequently quenched to room temperature. This treatment lead to a substantial volume increase and porosity of the pill. The chemical composition was determined by EDX which confirmed the composition Bi_12_Rh_3_Sn_3_I_9_ for microcrystalline powder as well as single crystals, except for a minor tin deficiency (Table 2).

**Table 2:** EDX data of the solid product obtained from a mixture of Bi, Rh, Sn, and BiI_3_ (12:3:3:9) that was heated at 310 °C for several days.

element	Measured [at.‐%]	Calculated [at.‐%]^[a]^
Bi	45(3)	44.4
Rh	12.4(5)	11.1
Sn	8.5(6)	11.1
I	34(2)	33.3

[a] Calculation with respect to Bi_12_Rh_3_Sn_3_I_9_.

**Figure 3:**
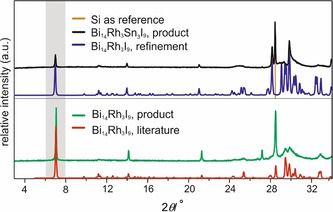
PXRD pattern (Cu${}_{K\alpha {}_{1}}$ , top) of the product of a mixture of Bi, Rh, Sn, and BiI_3_ (12:3:3:9) reacted at 310 °C (black), compared to the theoretical pattern (red) and measured pattern (green) of Bi_14_Rh_3_I_9_ as well as the refined pattern with TOPAS (blue).

**Figure 4:**
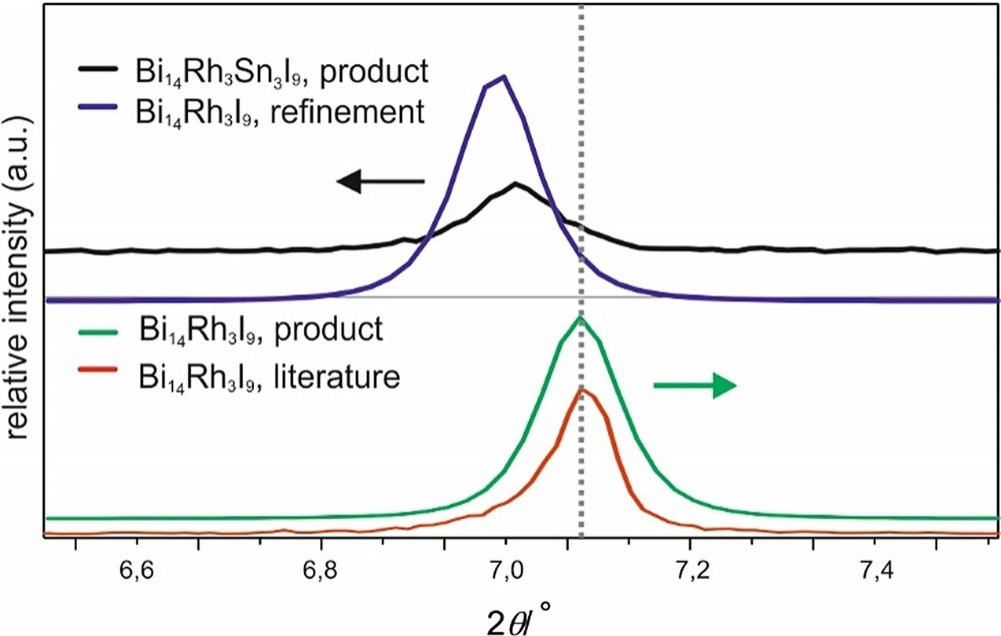
Inset of the PXRD pattern (Figure 3) shows shifted positions (distance from dotted line) of the new phase Bi_12_Rh_3_Sn_3_I_9_ towards smaller angles in contrast to Bi_14_Rh_3_I_9_.

The diffraction pattern of the product was very similar to a measured PXRD pattern of Bi_14_Rh_3_I_9_ (Figure 3), but the reflections were shifted toward lower 2*θ* angles. Using a silicon standard and the known crystal structure of Bi_14_Rh_3_I_9_, lattice parameters were determined by Rietveld refinement performed with TOPAS (Figure 4; Supporting Information Table 2). To solve the crystal structure of the new compound, crystals were grown and investigated with single‐crystal X‐ray diffraction.

### Crystal structure of Bi_12_Rh_3_Sn_3_I_9_

**Figure 5:**
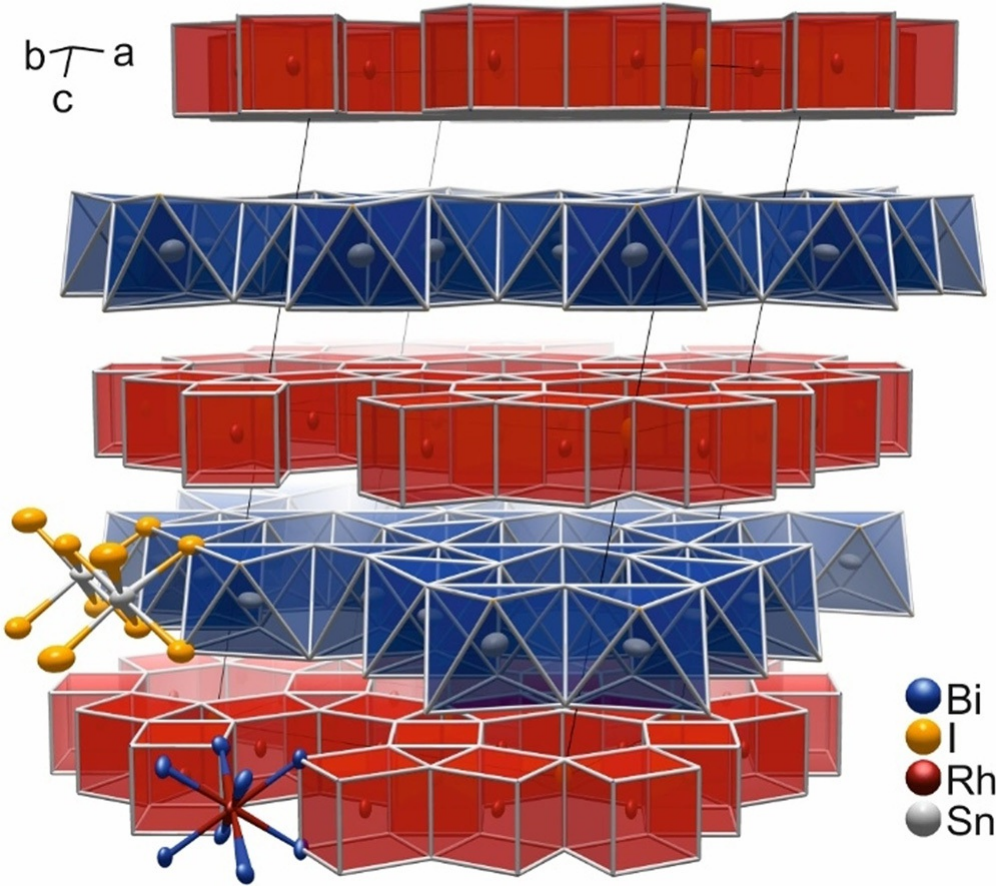
Crystal structure of Bi_12_Rh_3_Sn_3_I_9_. The intermetallic kagome network _∞_^2^[(Bi_4_Rh)_3_I]^2+^ (red) alternates with the anionic spacer layer _∞_^2^[Sn_3_I_8_]^2−^ (blue).

Considering the thermal studies and the Ostwald–Miers range, an optimized temperature program was deduced for the growth of black hexagonal‐shaped single‐crystals of Bi_12_Rh_3_Sn_3_I_9_. Since structural problems, for example, twinning and stacking disorder similar as in Bi_14_Rh_3_I_9_,^[17]^ can hamper the crystal structure refinements of the new compound, the synthesis was optimized to yield single‐crystals up to 400 μm. Structure determinations using those crystals confirmed the composition Bi_12_Rh_3_Sn_3_I_9_ and the substitution of bismuth(III) with tin(II) in the anionic spacer (Tables 3–5, Figure 5).

**Table 3:** Structural data for Bi_12_Rh_3_Sn_3_I_9_ opposed to the data of Bi_14_Rh_3_I_9_.^[17]^

	Bi_12_Rh_3_Sn_3_I_9_	Bi_14_Rh_3_I_9_
space group	*C*12/*m*1	*P* $\bar{1}$
*a* [Å]	15.8385(11)	9.1661(3)
*b* [Å]	9.1353(6)	15.8361(5)
*c* [Å]	12.9204(9)	14.2978(5)
*α* [°]	90	62.746(1)
*β* [°]	101.801(4)	80.922(2)
*γ* [°]	90	89.936(2)
cell volume [Å^3^]	1829.9(2)	1815.9(2)
*R*_1_[*F*_o_>4σ(*F*_o_)]	0.025	0.126
w*R*_2_ (all *F*_o_^2^)	0.058	0.069

**Table 4:** Coordinates and equivalent isotropic displacement parameters of the atoms in Bi_12_Rh_3_Sn_3_I_9_ at *T*=170(2) K. The occupancies of the positions Sn1 and Sn2 are 0.836(1) and 0.664(1), respectively.

site	Wyckoff position	*x*	*y*	*z*
Bi1	4*i*	0.30400(4)	1/2	0.12526(5)
Bi2	4*i*	0.23826(4)	0	0.12559(5)
Bi3	8*j*	0.37043(3)	0.17438(4)	−0.12562(4)
Bi4	8*j*	0.41234(3)	0.17438(4)	0.12522(4)
I1	2*a*	1/2	1/2	0
Rh1	4*e*	1/4	1/4	0
Rh2	2*b*	1/2	0	0
I2	4*i*	0.43862(8)	0	0.63369(10)
I3	8*j*	0.31085(5)	0.74538(9)	0.36545(7)
I4	4*i*	0.05471(9)	0	0.34294(11)
Sn1	4*h*	1/2	0.74020(16)	1/2
Sn2	4*i*	0.24486(12)	0	0.49193(15)

**Table 5:** Selected interatomic distances in Bi_12_Rh_3_Sn_3_I_9_.

Atom pair		*d* [Å]	Atom pair		*d* [Å]
Bi1‐Rh1	3x	2.8285(4)	Bi3‐Bi4	3x	3.4393(6)
Bi1‐Bi2	2x	3.1726(10)	Bi4‐Rh2	2x	2.8287(4)
Bi1‐Bi3	2x	3.1901(7)	Bi4‐Rh1	1x	2.8292(5)
Bi1‐Bi4	2x	3.4341(5)	Rh1‐Bi4	1x	2.8293(5)
Bi2‐Rh1	3x	2.8297(4)	Rh2‐Bi4	3x	2.8286(4)
Bi2‐Bi4	2x	3.1852(7)	I2‐Sn1	2x	3.2013(14)
Bi2‐Bi3	2x	3.4370(5)	I3‐Sn2	1x	3.1408(15)
Bi2‐Bi3	1x	3.4369(5)	I3‐Sn1	2x	3.1415(9)
Bi3‐Rh1	2x	2.8314(4)	I3‐Sn2	3x	3.1420(15)
Bi3‐Rh2	4x	2.8321(4)	I4‐Sn1	4x	3.2254(15)
Bi3‐Bi4	1x	3.1724(7)	I4‐Sn2	1x	3.226(2)
Bi3‐Bi3	2x	3.1861(8)	Sn1‐I2	2x	3.2014(14)
Bi3‐Bi1	1x	3.1900(7)	Sn2‐I3	2x	3.1407(15)

The crystal structure of Bi_12_Rh_3_Sn_3_I_9_ comprises intermetallic layers that are almost identical to the kagome layers found in Bi_14_Rh_3_I_9_.^[17]^ In contrast, a different anionic spacer is found in Bi_12_Rh_3_Sn_3_I_9_. In Bi_14_Rh_3_I_9_, the intermetallic layers are separated by iodido‐bismuthate _∞_^1^[Bi_2_I_8_]^2−^ zigzag chains, which can also be seen as a double layer of iodide ions in which half of the octahedral voids are filled by bismuth(III) cation. The sum formula can be structured as follows (with □ representing a void octahedral site) [Eq. 1]:(1)
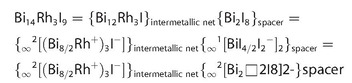



In contrast, the anionic spacer in Bi_12_Rh_3_Sn_3_I_9_ is an iodido‐stannate layer _∞_^2^[Sn_3_□I_8_]^2−^ (Figure 5), in which three quarters of the octahedral voids are occupied by tin(II) cations [Eq. 2]:(2)
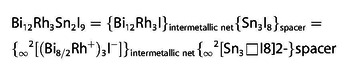



The substitution of two bismuth(III) cations by three tin(II) cations is isoelectronic and does not change the layer charges. The assigned oxidation states are supported by the valence sums *υ* of the cations, calculated based on bond‐length bond‐strength correlations:^[27]^ *υ*(Bi_spacer_)=2.99–3.04 for Bi_14_Rh_3_I_9_, and *υ*(Sn_spacer_)=1.89–1.99 for Bi_12_Rh_3_Sn_3_I_9_ (Supporting Information Table 3). The cross‐check with opposite assignments yields no meaningful results (Supporting Information Table 4).

In the crystal structure determination, the tin atoms appear to be disordered over all octahedral voids, yet not statistically with 75 % occupancy for all position. Instead, the occupancies are rather different and specialized with *occ*(Sn1)=83.6(1) %≈5/6 and *occ*(Sn2)=66.4(1) %≈2/3. A reasonable explanation is the following:

The structure shows a high degree of pseudo‐symmetry. The layer symmetry of the intermetallic layer is *P*6/*mmm*. Within the accuracy of the lattice parameters, |*c* ^.^ cos*ß*|/*a=*1/6, that is, in orthogonal projection on the (0 0 1) plane, every sixth unit cell exactly matches. Nonetheless, the symmetry of the structure is neither trigonal nor orthorhombic, because the position of the symmetry elements of the individual layers do not match in space. Thus, a larger unit cell with *c*’=6*c* ^.^ sin*ß* is not appropriate.

The _∞_^2^[Sn_3_I_8_]^2−^ spacer layer has a corrugated surface with I4 protruding by 0.29 Å from a plane defined by I2 and I3. I4 is positioned directly above and below I1, which resides in the hexagonal prismatic voids of the intermetallic network and corresponds to a “dent” in the surface of the latter (I1⋅⋅⋅I4=4.34 Å). Thereby the two types of layers interlock.

**Figure 6:**
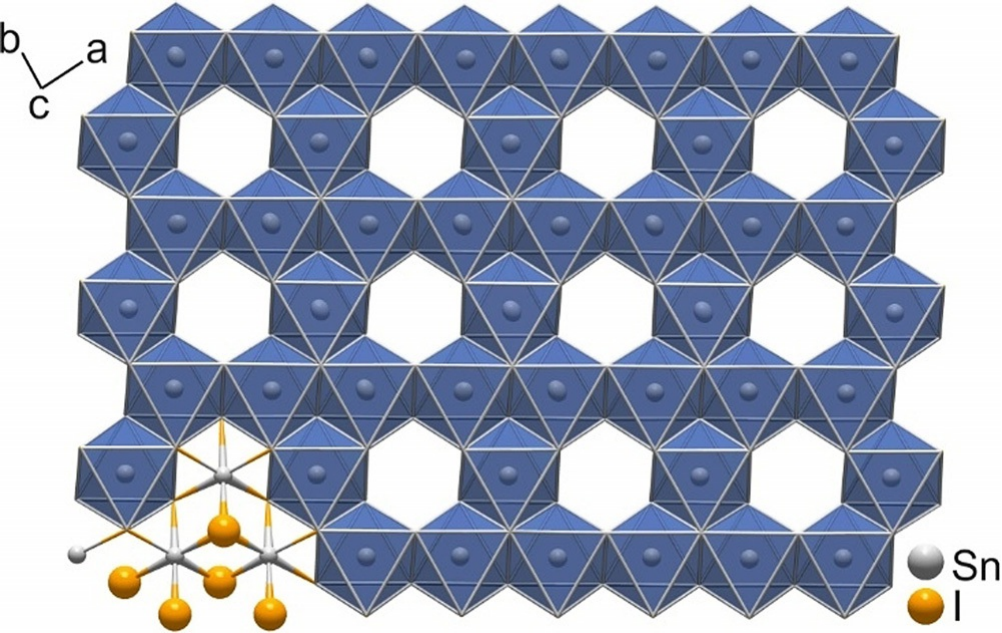
Anionic spacer layer _∞_^2^[Sn_3_I_8_]^2−^ in Bi_12_Rh_3_Sn_3_I_9_ with tin(II) cations forming a [3.6.3.6; 3^2^.6^2^] net.

The most common ordered arrangement associated with the average occupancy of 75 % is the kagome net, which belongs to the uniform tilings. It is represented by the Schläfli symbol [3.6.3.6] and has the hexagonal plane‐group symmetry *P*6*mm*. However, this does not match the occupancies of 5/6 for Sn1 and 2/3 for Sn2. A kagome‐type spacer layer with long‐range translational order along the stacking direction would correspond to *occ*(Sn1)=100 % and *occ*(Sn2)=50 %. Kagome‐type spacer layers that are ordered within each layer but show statistical disorder along the stacking direction would correspond to *occ*(Sn1)=*occ*(Sn2)=75 %. However, another cation distribution within the iodido‐stannate layer is consistent with the refinement results: the 2‐isogonal tiling with Schläfli symbol [3.6.3.6; 3^2^.6^2^] and rectangular plane‐group symmetry *P*2*mm*. Assuming ordered cation distribution in each layer but rotational disorder (120° and 240°) in the sequence of spacer layers, average occupancies of (100 %+75 %+75 %)/3=5/6 for Sn1 and (50 %+75 %+75 %)/3=2/3 for Sn2 result (Figure 6). As this suggests a locally ordered structure, we used the latter model for the electronic band structure calculations. The according symmetry reduction, however, could not be resolved in the X‐ray diffraction data because of missing long‐range order (orientational stacking faults of the spacer layers).

### Electronic structure of Bi_12_Rh_3_Sn_3_I_9_

The presence of crystallographic positions with partial occupancy as observed for the title compound requires appropriate structure models for the intended simulations. Here, two different models are applied:

**Model A**: The experimental structure as obtained from the present single‐crystal XRD data (see Table 3 and Table 4) is considered in this model, but with all crystallographic positions completely occupied. The observed partial occupancy of the two inequivalent Sn positions is modelled by appropriate reduction of the electron numbers using the virtual crystal approximation (VCA). Experimental occupancies are 83.6 % and 66.4 % for Sn1 (4*h*) and Sn2 (4*i*), respectively, providing in the mean 1.67 and 1.33 valence electrons at the respective position. Using VCA, Sn1 is replaced by the tin‐like pseudo‐atom _49.67_Sn, and Sn2 by _49.33_Sn. Here, the notation _*Z*_Sn means that the pseudo‐atom carries the nuclear charge *Z* and the same number of electrons, thus guaranteeing overall charge neutrality. The advantage of this model is that it allows the use of the experimental crystal structure of Bi_12_Sn_3_Rh_3_I_9_. Its disadvantage consists in ignoring any subtle effect of disordered vacancies at the Sn positions.

**Model B**: In this model, half of the Sn2 sites are left empty, according to a [3.6.3.6; 3^2^.6^2^] net of tin(II) cations. All other Sn positions are completely occupied. In this way, the model contains the correct number of Sn atoms and, thus, implies a decent description of interlayer bonding. The likely present local order of the material is described correctly but is supplemented by an ordered arrangement of the vacancies that is only indirectly supported by the X‐ray data. This causes a reduction of crystal symmetry, which is now described by the space group *C*1*m*1 (no. 8). An advantage of Model B is the possibility to account for local atomic relaxation, which is expected to be of importance in the neighborhood of the vacancy. The structure details of Model B without and with relaxation are shown in the Supporting Information (Supporting Table 5), where the latter was performed without any symmetry constraint (space group *P*1) but under preservation of size and shape of the unit cell. The largest shift, by about 0.25 Å, is observed for I7, which is one of the atoms next to the Sn‐vacancy.

**Figure 7:**
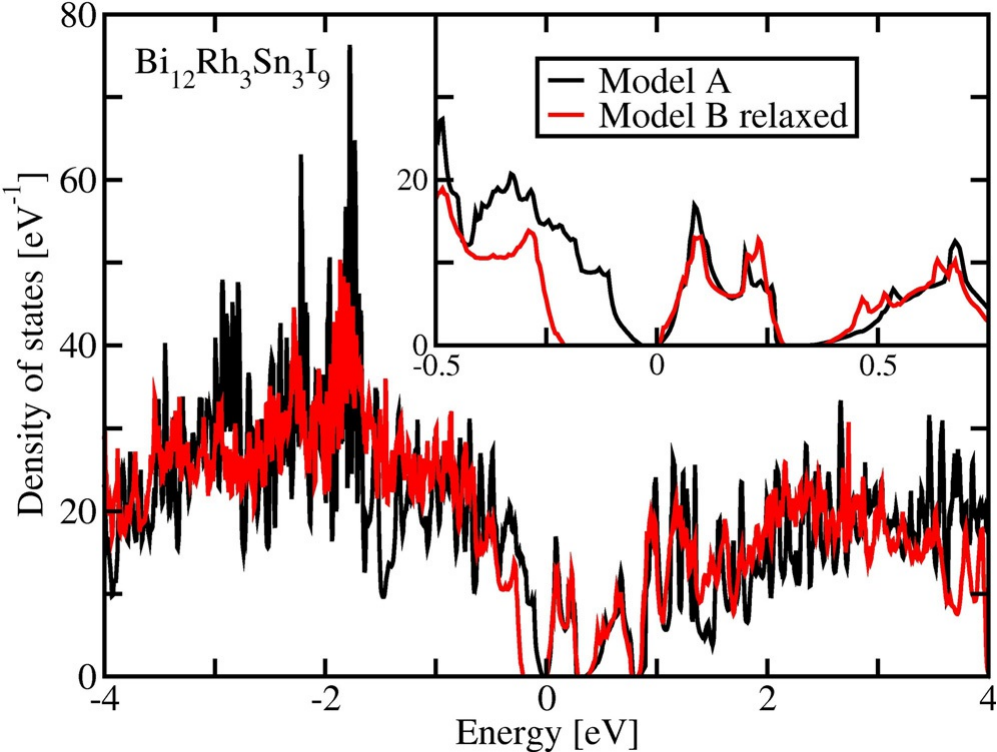
Density of states for two structure models of Bi_12_Rh_3_Sn_3_I_9._ The energy zero is placed at the bottom of the conduction band. The inset shows an enlarged view of the same data as the main figure.

DFT calculations were performed for both described structure models. Figure 7 shows a comparison of the total densities of states (DOS) of Model A and Model B.

For the latter, only results obtained after relaxation of the atomic positions are shown here and in the following. The overall DOS of both models agree well in the whole presented energy range (main figure), while differences are visible around the top of valence band/bottom of conduction band (inset). In particular, the size of the gap amounts to about 40 meV in Model A and to about 210 meV in Model B, while the relative positions and the shapes of the conduction bands up to 1 eV are similar in both models. Relaxation of the atomic positions of Model B has only a marginal effect on the gap size, which is enhanced by 10 meV compared with the unrelaxed structure. The dominant contributions to the total DOS at and around the Fermi level (*E*_F_) originate from the Bi 6p and Rh 4d states of the 2D TI layer, as had been found for Bi_14_Rh_3_I_9_. The spacer layer provides essential contributions to the total DOS of the valence band only below −0.5 eV and to the conduction band above +1.5 eV.

**Figure 8:**
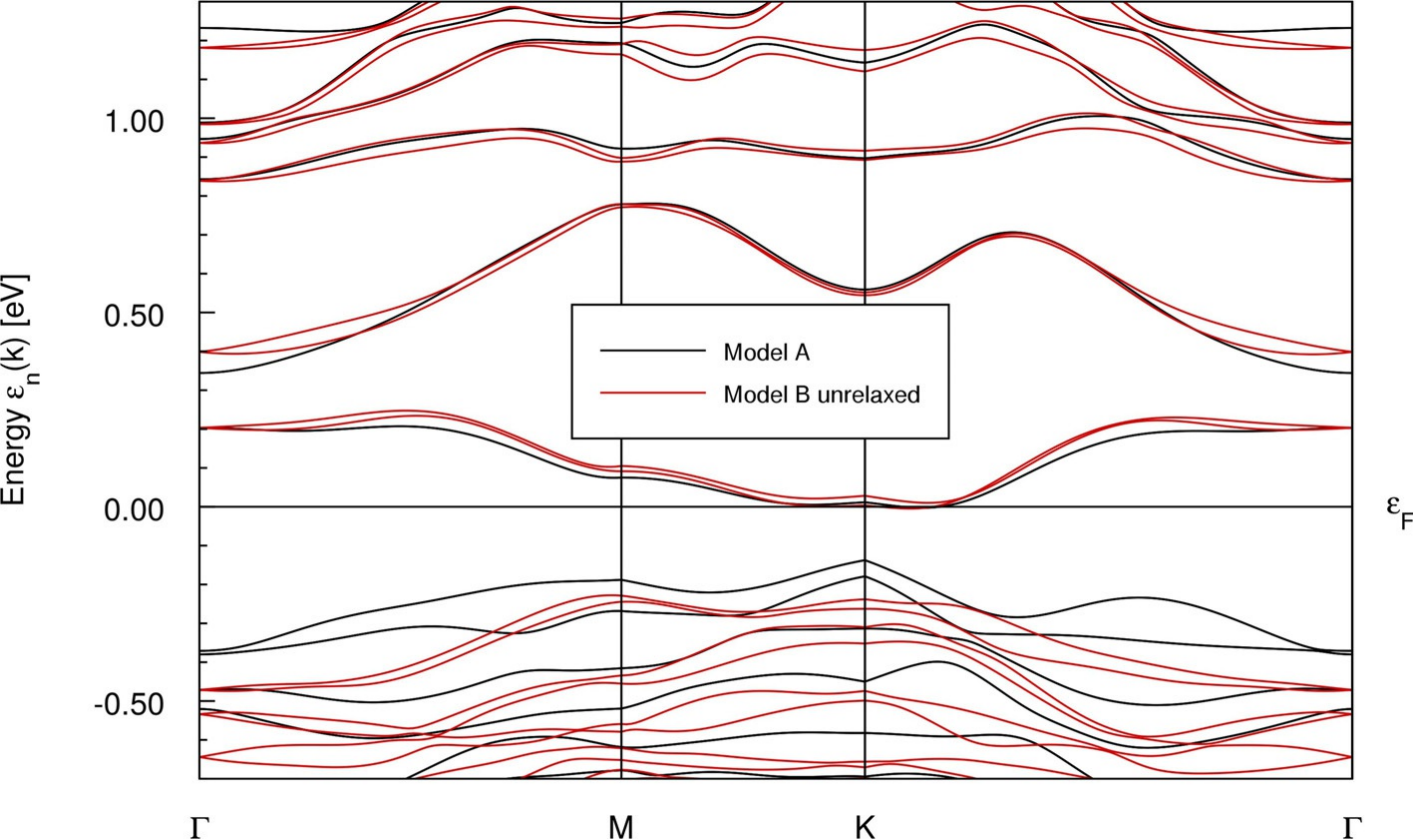
Band structure for two structure models of Bi_12_Rh_3_Sn_3_I_9_. The energy zero is placed at the bottom of conduction band.

Related band structures are presented in Figure 8. We limit this presentation to a pseudo‐hexagonal two‐dimensional (2D) Brillouin zone in the plane spanned by the primitive lattice vectors ***a*** and ***b***. This plane is parallel to the structural layers (Figure 4). We denote the reciprocal lattice vectors of the 2D lattice generated by ***a*** and ***b*** as a* and b*. The points M and K, with M=a*/2 and K=(a*+b*)/3, resemble symmetry points of a 2D hexagonal lattice but are not points of high symmetry in the present monoclinic (Model A) or triclinic (Model B) lattices. This is visible in Figure 8 at point K, where the dispersion shows small kinks.

As anticipated from the very similar DOS of both models, their band structures only differ in subtle details in the considered energy window close to the Fermi level. In particular, the four lowest conduction bands show almost the same dispersion in both models. The bands of Model A are two‐fold degenerate due to inversion symmetry, while related bands of Model B show small splitting. All bands close to the Fermi level show very small overall dispersion (<0.5 eV).

We conclude this section by stating that the electronic structures provided by Model A and Model B are qualitatively the same and both models undoubtedly yield an insulating ground state. The essential quantitative difference between both models consists in the size of the gap.

### *Z*_2_ invariants

For the considered structure model with centres of inversion, Model A, *Z*_2_ invariants were calculated through Fu–Kane indices.^[28]^ For the structure model without inversion centre, Model B, invariants were calculated according to Ref. [29]. This computation was carried out directly from the PW92 band structure without resorting to an approximate Wannier representation. For both cases, we find the invariants (*ν*_0_; *ν*_1_, *ν*_2_, *ν*_3_)=(0; 0, 0, 1), that is, the title compound is categorized as a weak topological insulator with the same invariants as the parent compound Bi_14_Rh_3_I_9_.^[9]^ The lowest gap within the conduction band carries the same topological properties as the fundamental gap, while the second lowest gap is trivial with (*ν*_0_; *ν*_1_, *ν*_2_, *ν*_3_)=(0; 0, 0, 0). We confirmed the automatized calculation of these invariants for the case of Model B by visual inspection of the Wannier centres.

The observed robustness of electronic structure with respect to structural details, together with the identification of identical sets of invariants in both models, provides confidence in the validity of the structure models for the determination of the topological properties.

### Transport measurements on Bi_12_Rh_3_Sn_3_I_9_ crystals

**Figure 9:**
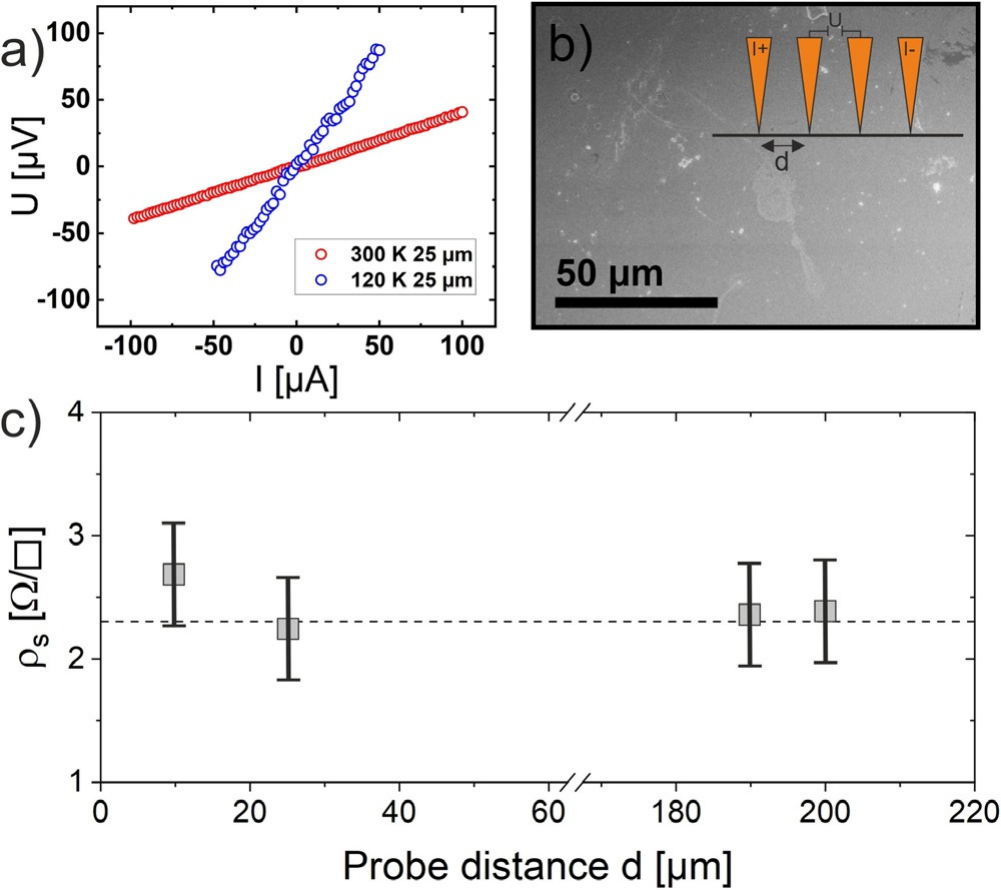
Resistivity of Bi_12_Rh_3_Sn_3_I_9_ measured by in situ four‐point probe technique. a) *U* ^*I*^ curves for fixed probe spacing at 120 and 300 K. b) SEM image of Bi_12_Rh_3_Sn_3_I_9_. The inset shows the collinear four‐point probe geometry. c) Averaged resistivity as a function of probe distance *d* measured at 300 K.

In order to also investigate electronic properties of this new TI compound experimentally, we performed in situ four‐point probe transport measurements. The measurements were made under ultra‐clean conditions, and the controlled in situ positioning of tungsten tips as on‐top contacts minimizes parasitic doping of the material. The linearity of the *U* ^*I*^‐curve clearly indicates metallic behaviour (Figure 9).

The (spatially averaged) resistivity of the material measured at 300 K is as low as 2.3 Ω □^−1^ and does not depend on the spacing *d* between the tips, which is indicative for a 2D transport channel. The resistivity measured at 120 K is slightly increased, but still metallic behaviour is obvious from the *U* ^*I*^‐curve, and thus activated transport along undoped bulk band channels plays only a minor role in this wide band gap TI material. Compared to previous transport studies on Bi_2_Te_2_Se,^[30]^ the conductivity is almost two orders of magnitude larger, despite the weak band dispersion shown in DFT. Whether this is induced by a percolated network of protected 1D edge channels or by a parasitic surface‐near doped bulk‐band channel needs to be investigated in future experiments.

## Conclusions

The exchange of bismuth(III) by tin(II) in the anionic spacer layer of Bi_14_Rh_3_I_9_ leads to the new weak 3D TI Bi_12_Rh_3_Sn_3_I_9_. Thereby, the iodido‐bismuthate chains are replaced by iodido‐stannate layers [Sn_3_I_8_]^2−^ while the intermetallic [(Bi_4_Rh)_3_I]^2+^ layers (2D TIs) are not subject to any modifications. DFT calculations also show a weak 3D TI character of the novel tin‐containing phase with a topological band gap of maximally 210 meV for a cation‐ordered model. The tin substitution leads to a slightly enlarged molar volume, but decreases the size of the topological band gap compared to Bi_14_Rh_3_I_9_. Nonetheless, layers with divalent cations are suitable spacers that efficiently prevent electronic coupling between the 2D TI layers. A thorough understanding of the crystal growth has benefited the synthesis of single crystals, which may be used for further physical studies, for example, ARPES. Furthermore, the results hitherto have shown that the composition of Bi_14_Rh_3_I_9_ can be altered not only theoretically,^[12]^ but indeed experimentally. In this respect, future research could emphasize on further modification of the anionic spacer by doping or intercalating other elements to generate new weak 3D TIs with adjusted band gaps. Of special interest would be to put magnetic cations in the spacer layer, which would be another step towards envisioned applications in low‐energy spintronics and quantum computing.

## Experimental Section

All starting materials and products were handled in an argon‐filled glove box (MBRAUN; *p*(O_2_)/*p*_0_<1 ppm, *p*(H_2_O)/*p*_0_ <1 ppm). Rhodium (Rh, >99.9 %) and bismuth (Bi, >99.9 %) were purchased from Merck. Bismuth was treated at 220 °C in a hydrogen flow to remove oxygen impurities. Bismuth(III)‐iodide (BiI_3_, >99.9 %) was bought from Sigma Aldrich and sublimated at 200 °C. Tin (Sn, >99.9 %) was purchased from Merck Millipore and used without further treatment. The quality of the starting materials was evaluated by X‐ray diffraction and EDX studies.

**Synthesis of Bi_12_Rh_3_Sn_3_I_9_ powder and crystals**: Based on the results of thermal analyses, a phase‐pure microcrystalline powder of Bi_12_Rh_3_Sn_3_I_9_ was synthesized by annealing a stoichiometric mixture of bismuth, rhodium, and bismuth triiodide at 310 °C for five days. The starting materials were ground in a ball mill for about 25 minutes (Pulverisette 23, Fritsch), pressed to pellets (msscientific, diameter 6–8 mm) to ensure maximum homogeneity, and sealed in a 3 mL evacuated silica ampule. After the heat treatment, the ampule was cooled to room temperature at a rate of −5 K min^−1^. The growth of larger Bi_12_Rh_3_Sn_3_I_9_ crystals was carried out in analogy to the procedure used for Bi_14_Rh_3_I_9_.^[17]^ A stoichiometric mixture of bismuth, rhodium, tin and bismuth triiodide was sealed in a 3 mL evacuated silica tube and heated from room temperature to 720 °C with a rate of 12 K min^−1^. The sample was held at 720 °C for at least 10 minutes, followed by cooling to 420 °C at the rate of −2 K min^−1^, where the temperature was kept for further 20 minutes. The process was continued by cooling to 310 °C at the rate of −1 K h^−1^ and annealing at this temperature for approximately 7 days. In the end, the ampule was quenched in cold water.

**Thermal analysis**: Differential Scanning Calorimetry (DSC) was performed to investigate formation and decomposition processes in the quaternary Bi‐Rh‐Sn‐I system. The starting materials in the desired ratio were sealed in a tiny silica ampule. The measurements were conducted with this ampule under equilibrium pressure using a DTA‐DSC Labsys TMA System (Setaram). The mixture was heated up to 800 °C and cooled down to room temperature with a rate of 2 or 5 K min^−1^.

**Powder and single‐crystal X‐ray diffraction**: Powder X‐ray diffraction (PXRD) data (Cu${}_{K\alpha {}_{1}}$ , *λ*=1.54059  Å, *T=*296(1) K) were collected using either an X'Pert Pro diffractometer (PANalytical, Bragg–Brentano geometry, Ge(2 2 0) hybrid monochromator, fixed divergence slits, PIXcel detector) or a Stadi P diffractometer (Stoe & Cie, Debye–Scherrer geometry, Ge(1 1 1) monochromator, Dectris Mythen 1 K strip detector). The samples for PXRD measurements were ground and fixed on a single‐crystal silicon sample holder. The Rietveld‐refinement was performed with the program TOPAS[^31^, ^32^] on a mixture of the target phase with Si standard. The references were extracted from the ICSD. Single‐crystal X‐ray diffraction (SCXRD) data were obtained using an Apex‐II kappa CCD diffractometer (Bruker) or imaging plate diffractometer IPDS‐II (STOE); Mo_Kα_ radiation, *λ*=0.71073 Å, *T=*170(2) K. Numerical absorption corrections were applied based on optimized crystal descriptions. The structure was solved with direct methods and subsequent refinements against *F*_o_^2^. Graphics of the crystal structures were developed with Diamond.^[33]^ Deposition Number 1989503 contains the supplementary crystallographic data for this paper. These data are provided free of charge by the joint Cambridge Crystallographic Data Centre and Fachinformationszentrum Karlsruhe Access Structures service www.ccdc.cam.ac.uk/structures.

**Scanning electron microscopy and energy‐dispersive X‐ray spectroscopy**: Scanning electron microscopy (SEM) (*U*_a_=5–15 kV) was performed using a SU8020 electron microscope (Hitachi) equipped with multi detector system for secondary and low‐energy backscattered electrons, while an Oxford Silicon Drift Detector X‐Max^N^ was used for the semi‐quantitative energy‐dispersive X‐ray spectroscopy^[34]^ (EDX) (*U*_a_=20 kV). To acquire electron images, the powders were fixed on a carbon pad settled on an aluminum sample holder. To obtain the average composition by EDX, pellets of the powder (6–8 mm in diameter) were pressed and embedded into EpoThin^TM2^ epoxy resin (Buehler) and epoxy hardener (Buehler) under vacuum. After grinding the embedded pellets with a MetaServ 250 (Buehler, silicon carbide grinding paper) and subsequent polishing with a VibroMet^2^ (Buehler, MasterPrepTM alumina suspension), the surfaces were coated with carbon in an automatic rotary‐pump coating system (Quorum Q150R ES). To collect EDX data of a single crystal, crystals were fixed directly on a carbon film.

**In‐situ surface transport experiments**: Four‐point probe transport experiments were performed under ultra‐high vacuum conditions at room temperature and 120 K (liquid nitrogen) by means of a four‐tip scanning tunnelling microscope (STM) system (Omicron nanoprobe system) using NaOH‐etched W tips with typical radii of 100 nm. Flakes of the samples (about 200 μm thickness) were mounted on a transferable sample plate. The W tips were navigated and positioned individually in the field of view of a SEM across the sample. This allows various probe geometries and defined probe spacings. The resistance values were corrected to calculate the conductivity of the sample. The resistivity was measured at various positions and probe currents (1—100 μA) in order to average out the effect of chemical inhomogeneities, which were partly seen in SEM. More details about surface sensitive four‐point probe measurements can be found in Ref. [35].

**Computational methods**: DFT based calculations were performed with the full‐potential local‐orbital (FPLO) code,^[36]^ version 18.00—52.^[37]^ The exchange and correlation energy was considered in the local density approximation with PW92 parameterization.^[38]^ Optionally, optimization of internal atomic coordinates was performed in scalar relativistic mode unless the forces on individual atoms fell below 20 meV Å^−1^. The final self‐consistent calculations of the charge density were carried out using the four‐component full‐relativistic mode of FPLO. This effort is necessary due to the sizable spin‐orbit coupling of all elements of the considered compound. The following basis states were treated as valence states (default FPLO basis set): Bi: 5s, 5p, 5d, 6s, 7s, 6p, 7p, 6d; Rh: 4s, 4p, 5s, 6s, 4d, 5d, 5p; Sn and I: 4s, 4p, 4d, 5s, 6s, 5p, 6p, 5d. The ***k***‐space integrals were evaluated with the linear tetrahedron method using a ***k***‐mesh with 12×12×7 intervals in the full Brillouin zone for all calculations.

## Conflict of interest

The authors declare no conflict of interest.

## Supporting information

As a service to our authors and readers, this journal provides supporting information supplied by the authors. Such materials are peer reviewed and may be re‐organized for online delivery, but are not copy‐edited or typeset. Technical support issues arising from supporting information (other than missing files) should be addressed to the authors.

Supplementary
