# Supplementary material for: The Weak 3D Topological Insulator Bi12Rh3Sn3I9
Source: Chemistry. 2020 Oct 4;26(67):15549–57. doi: 10.1002/chem.202001953 (PMC7756808; doi:10.1002/chem.202001953)
Supplement: Supplementary file 1 — Supplementary [file CHEM-26-15549-s001.pdf]

Chemistry–A European Journal

Supporting Information

The Weak 3D Topological Insulator $Bi_{12}Rh_3Sn_3I_9$

Mai Lê Anh,[a] Martin Kaiser,[a] Madhav Prasad Ghimire,[b, c] Manuel Richter,[c, d] Klaus Koepernik,[c] Markus Gruschwitz,[e] Christoph Tegenkamp,[e] Thomas Doert,[a] and Michael Ruck*[a, f]

Synthesis of $Bi_{12}Rh_3Sn_3I_9$ polycrystalline powder

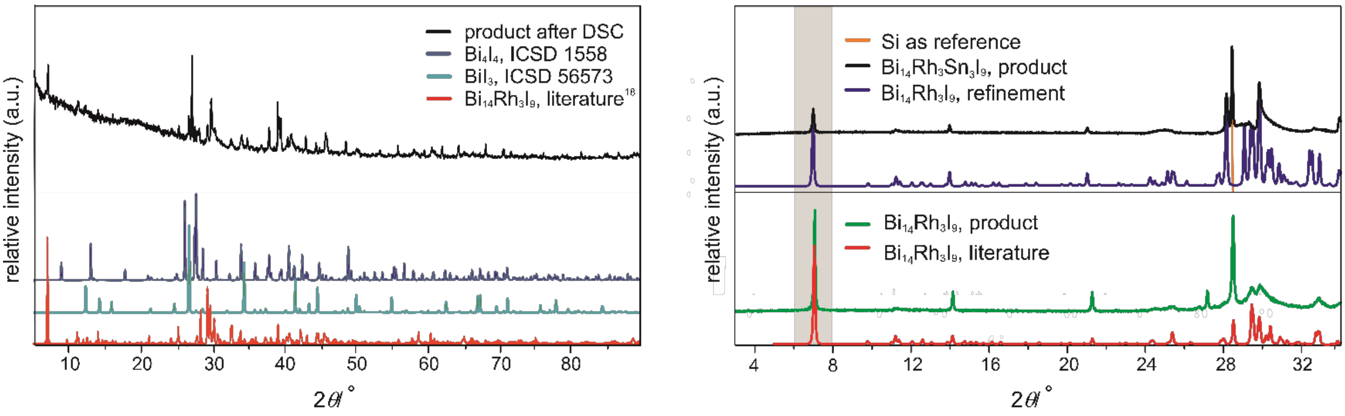

Supporting 1. Left: Diffraction pattern (Cu-Kα_1) of the heated DSC mixture (left) compared to the diffraction patterns of $Bi_{14}Rh_3I_9$, Bi_4I_4 and BiI_3. Right: Diffraction pattern (right) of phase pure $Bi_{12}Rh_3Sn_3I_9$.

Comparison of structural data of $Bi_{12}Rh_3Sn_3I_9$ and $Bi_{14}Rh_3I_9$.[18]

Supporting 2: Structural data of $Bi_{12}Rh_3Sn_3I_9$ derived from the Rietveld refinement compared to the reported structural data of $Bi_{14}Rh_3I_9$.

	$Bi_{12}Rh_3Sn_3I_9$	$Bi_{14}Rh_3I_9$	rel. deviation/%
space group	*C*12/*m*1	$P\bar{1}$	–
a/Å	15.8385(11)	9.1661(3)	–0.17
b/Å	9.1353(6)	15.8361(5)	+ 0.06
c/Å	12.9204(9)	14.2978(5)	+ 0.39
α/°	90	62.746(1)	+ 1.40
β/°	101.801(4)	80.922(2)	+ 0.20
γ/°	90	89.936(2)	+ 0.09
cell volume/Å^3	1829.9(2)	1815.9(2)	+ 1.13
$R_1[F_o>4\sigma(F_o)]$	0.025	0.126	–
wR_2 (all F_o^2)	0.058	0.069	–

Supporting 3: Synopsis of the valence sum calculation for $Bi_{12}Rh_3Sn_3I_9$ [35]. The calculated valence sums for tin are 1.888–1.987, in accordance with tin(II).

Atom pair		*d*(Sn–I)/Å	*u*(Sn–I)	Assumed Bi: *u*(Bi–I)
Sn1 – I2	2x	3.2014	0.303	0.377
Sn1 – I3	2x	3.1415	0.357	0.443
Sn1 – I4	2x	3.2254	0.284	0.353
valence sum			1.888	2.346
Atom pair		*d*(Sn–I)/Å	*u*(Sn–I)	Assumed Bi: *u*(Bi–I)
Sn2 – I2	1x	3.2360	0.276	0.343
Sn2 – I3	2x	3.1420	0.356	0.442
Sn2 – I3	2x	3.1407	0.357	0.444
Sn2 – I4	1x	3.2256	0.284	0.353
valence sum			1.986	2.468

Supporting 4: Synopsis for the valence sum calculation for $Bi_{14}Rh_3I_9$. The calculated valence sums for bismuth are 2.987–3.039, in accordance with bismuth(III).

Atom pair		*d*(Bi–I)/Å	*υ*(Bi–I)	Assumed Sn: *υ*(Sn–I)
Bi13 – I2	1x	3.119	0.470	0.379
Bi13 – I2	1x	3.365	0.242	0.195
Bi13 – I3	1x	2.905	0.839	0.676
Bi13 – I4	1x	2.921	0.803	0.647
Bi13 – I5	1x	3.124	0.464	0.374
Bi13 – I6	1x	3.400	0.220	0.177
valence sum	1x		3.039	2.448
Atom pair		*d*(Bi–I)/Å	*υ*(Bi–I)	Assumed Sn: *υ*(Sn–I)
Bi14 – I5	1x	3.312	0.279	0.225
Bi14 – I6	1x	3.152	0.430	0.347
Bi14 – I7	1x	2.944	0.755	0.608
Bi14 – I8	1x	2.939	0.765	0.616
Bi14 – I9	1x	3.090	0.509	0.410
Bi14 – I9	1x	3.355	0.249	0.200
valence sum			2.987	2.406

Supporting 5: Details of Model B (without relaxation: space group no. 8, *C*1*m*1; with relaxation: space group no. 1, *P*1). Lattice constants and angles for the case without relaxation are identical to those given in Table 3. For the case with relaxation, $\tilde{a} = \tilde{b}$ = 9.14210 Å, axis angles are 79.80˚, 100.20˚, and 120.05˚, and the Cartesian $\tilde{x}$ - $\tilde{y}$ plane is rotated with respect to the *x* - *y* plane (*z* and *c* are unchanged).

Positions		Without relaxation			With relaxation		
		x/a	*y/b*	*z/c*	*x/a*	*y/b*	*z/c*
Bi1	2a	-0.19600	0	0.12526	-0.196	0.196	0.125
Bi2	2a	0.19600	0	-0.12526	0.197	-0.197	-0.128
Bi3	2a	0.23826	0	0.12559	0.237	-0.237	0.123
Bi4	2a	-0.23826	0	-0.12559	-0.239	0.239	-0.124
Bi5	4b	-0.12957	-0.32562	-0.12562	–0.455<br>0.196	–0.196<br>0.455	–0.121<br>–0.121
Bi6	4b	0.12957	-0.32562	0.12562	–0.197<br>0.456	–0.456<br>0.197	0.127<br>0.127
Bi7	4b	-0.08766	-0.32562	0.12522	–0.416<br>0.239	–0.239<br>0.416	0.128<br>0.128
Bi8	4b	0.08766	-0.32562	-0.12522	–0.237<br>0.414	–0.414<br>0.237	–0.122<br>–0.122
I1	2a	0.00000	0	0.00000	0.001	-0.001	0.008
Rh1	4b	0.25000	0.25000	0.00000	0.500<br>–0.000	0.000<br>–0.500	0.001<br>0.001
Rh2	2a	0.50000	0	0.00000	-0.500	0.500	0.003
I2	2a	0.43862	0	-0.36631	0.439	-0.439	-0.361
I3	2a	-0.43862	0	0.36631	-0.445	0.445	0.362
I4	4b	-0.18915	0.24538	0.36545	0.062<br>–0.438	0.438<br>–0.062	0.362<br>0.361

I5	4b	0.18915	0.24538	-0.36545	0.439<br>–0.056	0.056<br>–0.439	–0.362<br>–0.362
I6	2a	0.05471	0	0.34294	0.057	-0.057	0.349
I7	2a	-0.05471	0	-0.34294	-0.039	0.039	-0.342
Sn1	4b	0.00000	0.24020	0.50000	0.242<br>–0.239	0.239<br>–0.242	–0.497<br>–0.497
Sn2	2a	0.24486	0	0.49193	0.247	-0.247	0.496
